# Approach to Management of Thrombotic Thrombocytopenic Purpura at University of Cincinnati

**DOI:** 10.1155/2013/195746

**Published:** 2013-12-16

**Authors:** N. Abdel Karim, S. Haider, C. Siegrist, N. Ahmad, A. Zarzour, J. Ying, Z. Yasin, R. Sacher

**Affiliations:** ^1^Department of Internal Medicine, University of Cincinnati College of Medicine, Cincinnati, OH 45267, USA; ^2^Division of Hematology and Oncology, University of Cincinnati College of Medicine, 231 Albert Sabin Way, Cincinnati, OH 45267, USA; ^3^Department of Environmental Health, University of Cincinnati College of Medicine, Cincinnati, OH 45267, USA; ^4^Department of Hematology and Oncology, Baylor College of Medicine, Houston, TX 76706, USA; ^5^Hoxworth Blood Center, University of Cincinnati College of Medicine, Cincinnati, OH 45267, USA

## Abstract

Thrombotic Thrombocytopenic Purpura (TTP) is a rare hematologic emergency, congenital or acquired, characterized by ischemic damage of various organs because of platelet aggregation. It is the common name for adults with microangiopathic hemolytic anemia, thrombocytopenia, with or without neurologic or renal abnormalities, and without another etiology; children without renal failure are also described as TTP. Plasma exchange (PE) is the main stay of treatment in combination with steroids and immunosuppressive therapies. The monoclonal antibody against CD20 Rituximab decreases the production of antibodies from B lymphocytes and it is used for antibodies-mediated diseases including TTP. We present our data on retrospective analysis of rituximab in treatment of TTP at University of Cincinnati in a series of 22 patients from 1997 to 2009. Our results showed that PE with immunosuppressive therapy resulted in decreased duration of PE, relapse rate, and increased duration of remission in patients with TTP.

## 1. Introduction

TTP is a rare hematologic emergency in which various organs, mainly the brain and kidneys, are affected by ischemic damage due to platelets aggregations. It is characterized by thrombocytopenia, MAHA, fever, and neurological and renal abnormalities; however, this pentad is not necessary for diagnosis. TTP may be congenital or acquired as a result of HIV, connective tissue disorder, cancers, drugs like quinine, mitomycin C, cyclosporine, oral contraceptives, and ticlopidine or it may be idiopathic. Only thrombocytopenia and MAHA without another clinically apparent etiology (e.g., disseminated intravascular coagulation, malignant hypertension, severe preeclampsia, sepsis, and systemic malignancy) are required to suspect the diagnosis of TTP and to initiate PE. MAHA is defined as nonimmune hemolysis (i.e., negative direct antiglobulin test) with prominent red cell fragmentation (schistocytes) observed on the peripheral blood smear.

The pathogenesis may be autoimmune in nature since autoantibodies against ADAMTS13 (acronym for a Disintegrin and a Metalloproteinase with Thrombospondin-1 Motifs, 13th member of the family), which cleaves von Willebrand Factor (vWF), are typically present in most cases of idiopathic TTP. These antibodies cause the absence of ADAMTS 13 protease activity and the persistence of vWF. Subsequently the procoagulation tendency dominates and causes the systemic abnormalities. The mainstay of treatment for patients with TTP is PE in conjunction with steroids. The mortality rate of TTP prior to the use of PE was approximately 90 percent [1–3] and is currently 20 percent or less in patients treated with PE [3–5]. PE reverses the platelet consumption responsible for the thrombus formation and symptoms in TTP.

Although the majority of patients with TTP achieve remission with PE + steroids therapy [6], more than one-third of the patients survive the acute phase relapse within 10 years [7]. Different immunosuppressive therapies (such as intravenous immunoglobulins, vincristine, cyclophosphamide) [8–11] and splenectomy [12] have been suggested with no definitive benefit.

Rituximab is a monoclonal antibody directed against CD20 which is specific to B lymphocytes. It depletes the production of antibodies from these lymphocytes and thus has been used for antibodies-mediated diseases including TTP. Here we report our experience at the University of Cincinnati for over a decade of using Rituximab in the treatment of TTP patients.

## 2. Aims and Methodology 

The objective of this study was to review the medical records of patients diagnosed with TTP at the University of Cincinnati between the period of 1997 and 2009 and compare the outcome of patients who received PE alone to those who were treated with PE in combination with Rituximab-based chemotherapy (PE + R/RC). The variables reviewed were patient's demographics, types of treatment received (i.e., PE alone versus PE + R/RC), duration of PE, remission rate, and duration of remission. IRB approval was obtained and patient's outcome was followed during this period of time. Rituximab was added to the treatment if there is no response after 4 weeks of PE or there is brief response with relapse in 4 weeks. It was given at 375 mg/sq. meter every week for four doses.

## 3. Statistical Analysis 

Numerical and categorical variables were summarized using median (range) and frequency (in %), respectively. Nonparametric Wilcoxon rank sum tests were used to compare medians between groups while frequencies were compared using Fisher's exact test. For patients in the PE + R/RC group, their duration time using PE only was compared to that of PE and R/RC combined using a Wilcoxon signed-rank test. All patients were followed up to their last visit or death after treatment. Survival curves were estimated and plotted using a Kaplan-Meier survival method and compared between PE and PE + R/RC groups using a log rank test. All statistical analyses were performed using a SAS 9.2 (SAS, Cary, NC) package. *P* values <0.05 were considered statistically significant.

## 4. Results 

A total of 22 patients were studied. The median (range) of age was 41.5 (17 to 61) and the female : male ratio was 19 : 3. Thirteen patients (59%) were treated with PE only while the rest of 9 patients (41%) were treated with PE + R/RC. Please see Table 1. All patients in the PE + R/RC group were female. Among the rest of 10 female patients in the PE group, 3 were found pregnant. All patients started the treatment at the time of diagnosis, only one patient started the next day because of issues with the functioning of line. Patient's baseline clinical characteristics (presence of proteinuria, presence of schistocytes on blood smear, white blood cells count, hemoglobin levels, reticulocytes count, creatinine levels, and LDH levels) showed no difference between the two groups.

The median (range) of duration of PE was 284 (53, 337) days in the PE group. In the PE + R/RC group, the median (range) of duration using Rituximab only (R/RC only) was 151 (30, 291) days, shorter than that of the PE group (*P* = 0.0912). However, the entire duration of PE in the PE + R/RC group was 220 (69, 624) days, which showed no difference to the duration in the PE group (*P* = 0.742).

It is important to underline this point because the decrease in duration of PE reflects a faster achievement of remission of the disease. Although PE is known to decrease the mortality rate of TTP from 90 percent (prior to the use of PE) to 20 percent, the procedure itself may have adverse reactions, such as pneumothorax, hemorrhage, local and systemic infection at catheter site, venous thrombosis, catheter obstruction, citrate anticoagulant-induced symptoms of hypocalcemia, (paresthesias, muscle cramps, nausea and vomiting, hypotension, and tetany), allergic symptoms including anaphylactoid reactions, or transfusion-related acute lung injury (TRALI) [13].

**Figure 1:**
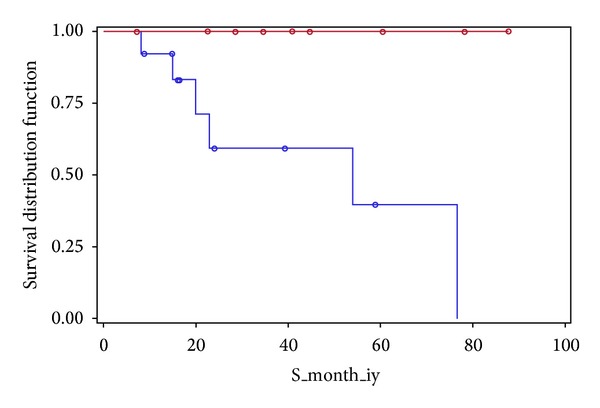
Survival curves of PE + R/C (red) versus PE (blue) alone arm.

All patients in the PE + R/RC were followed by a median (range) of 41 (7, 88) months and all survived. Patients in the PE group were followed by 20 (8, 77) months and 6 (46.2%) died after treatment. From the review of charts, we were able to identify the cause of death as intracranial hemorrhage in 1 patient and five deaths were attributed to catheter-related sepsis. Thus the death rate was higher in the PE group (*P* = 0.046) and underlines the importance of immunosuppression using rituximab in the treatment of TTP. Nevertheless, the 3 pregnant women in the PE group were all alive.  Figure 1 shows survival curves in the two groups. The PE group showed a lower survival curve than that of the PE + R/RC group, which was a flat line at 100% given that all survived up to the last visit (*P* = 0.011).

## 5. Discussion 

There is rationale for the use of rituximab in patients with TTP who do not respond promptly to PE and steroids or who have a relapse. Such patients almost always have severe ADAMTS13 deficiency and a demonstrable inhibitor. We were not able to find ADAMTS 13 values from retrospective analysis of the charts. However, this rationale is not often present during their first episode, as information concerning ADAMTS13 activity is generally not available at the time PE is instituted. Patients with a more severe course and more neurologic abnormalities, who either do not respond to PE, develop worsening disease in spite of continuing PE plus glucocorticoids, or have relapsing disease, may benefit from more intensive immunosuppressive treatment.

Rituximab can be administered during a course of PE. The dose of rituximab should be given immediately after the apheresis procedure to avoid unnecessary removal of the antibody. Although plasmapheresis removes much of the rituximab, PE on the day following rituximab treatment does not appear to impair rituximab's effectiveness [14]. This may be because the standard rituximab dose used (375 mg/m^2^) may exceed the dose required to deplete autoantibody-producing B cells. No increase in infections was documented during the first year of follow-up in the rituximab-treated group.

On review of the literature, Goyal et al. showed that 4 out of 12 patients (33%) who received PE and rituximab relapsed after 62 ± 8.5 months achieving remission [15].

There are no randomized trials evaluating the benefit of combining rituximab with PE. However, observational studies have suggested good outcomes in some settings. Rituximab should be considered in the management of TTP along with PE and well-designed prospective studies are needed to evaluate its role in TTP.

## 6. Conclusion 

TTP is adequately treated with PE in the acute setting; however, PE with immunosuppressive therapy trending towards a decreased duration of PE, relapse rate, and increased duration of remission. Prospective studies with immunosuppressive therapy upfront are needed to substantiate this.

## Figures and Tables

**Table 1:** Characteristics of patients in PE and PE + R/C arm.

Variable	Category	All (*N* = 22)	PE (13)	PE + R/C (9)	*P* value
Age		41.5	46	38	0.3771
Gender	Female	19 (86.4%)	10 (76.9)	9 (100)	0.2403
Race	African-American	16 (72.7%)	8 (61.5%)	8 (88.9%)	0.3330
New/Relapsed	Relapsed	5 (22.7%)	1 (7.7%)	4 (44.4%)	0.1159
Pregnancy	Yes	3 (13.6%)	3 (23.1%)	0 (0%)	0.2403
Schistocytes	Present	21 (95.5%)	12 (92.3%)	9 (100%)	1.000
Proteinuria	Yes	9 (40.9%)	4 (30.8%)	5 (55.6%)	0.3842
Died		6 (27.3%)	6 (46.2%)	0 (0%)	0.0461
Platelets (×1000)		15.5 (0.03, 60)	21 (4, 60)	8 (0.03, 27)	0.0839
WBC		10.4 (3.7, 16.4)	10.8 (4.1, 10.8)	8.9 (3.7, 16.4)	0.6706
Hb		8.5 (4.4, 10.7)	8.8 (6.4, 10.2)	7.4 (4.4, 10.7)	0.3602
Reticulocytes		4.9 (3.0, 12.3)	4.0 (3.0, 12.3)	7.1 (4.2, 11.5)	0.3624
Creatinine		1.0 (0.04, 9.8)	1.4 (0.4, 9.8)	1.0 (0.5, 1.9)	0.2018
LDH		827.5 (225, 2437)	1000 (225, 2302)	698 (226, 2437)	0.7283
Duration of PE		252 (53, 2624)	284 (53, 2337)	220 (69, 2624)	0.7418
Time between 1st PE and 1st Ritual		—	—	69 (0, 2375)	

## References

[1] Amorosi EL, Ultmann JE (1966). Thrombotic thrombocytopenic purpura: report of 16 cases and review of the literature. *Medicine*.

[2] Remuzzi G, Garella S (1987). HUS and TTP: variable expression of a single entity. *Kidney International*.

[3] von Baeyer H (2002). Plasmapheresis in thrombotic microangiopathy-associated syndromes: review of outcome data derived from clinical trials and open studies. *Therapeutic Apheresis*.

[4] Kremer Hovinga JA, Vesely SK, Terrell DR, Lämmle B, George JN (2010). Survival and relapse in patients with thrombotic thrombocytopenic purpura. *Blood*.

[5] Rock GA, Shumak KH, Buskard NA (1991). Comparison of plasma exchange with plasma infusion in the treatment of thrombotic thrombocytopenic purpura. *The New England Journal of Medicine*.

[6] Balduini CL, Gugliotta L, Luppi M (2010). High versus standard dose methylprednisolone in the acute phase of idiopathic thrombotic thrombocytopenic purpura: a randomized study. *Annals of Hematology*.

[7] Shumak KH, Rock GA, Nair RC (1995). Late relapses in patients successfully treated for thrombotic thrombocytopenic purpura. *Annals of Internal Medicine*.

[8] Durand JM, Lefevre P, Kaplanski G, Soubeyrand J (1993). Ineffectiveness of high-dose intravenous gammaglobulin infusion in thrombotic thrombocytopenic purpura. *American Journal of Hematology*.

[9] Durand JM, Lefevre P, Kaplanski G, Telle H, Soubeyrand J (1992). Vincristine for thrombotic thrombocytopenia purpura. *The Lancet*.

[10] O’Connor NTJ, O’Shea MJ, Hill LF (1992). Vincristine for thrombotic thrombocytopenic purpura. *The Lancet*.

[11] Udvardy M, Rak K (1990). Cyclophosphamide for chronic relapsing thrombotic thrombocytopenic purpura. *The Lancet*.

[12] Crowther MA, Heddle N, Hayward CPM, Warkentin T, Kelton JG (1996). Splenectomy done during hematologic remission to prevent relapse in patients with thrombotic thrombocytopenic purpura. *Annals of Internal Medicine*.

[13] Rizvi MA, Vesely SK, George JN (2000). Complications of plasma exchange in 71 consecutive patients treated for clinically suspected thrombotic thrombocytopenic purpura-hemolytic-uremic syndrome. *Transfusion*.

[14] McDonald V, Manns K, Mackie IJ, Machin SJ, Scully MA (2010). Rituximab pharmacokinetics during the management of acute idiopathic thrombotic thrombocytopenic purpura. *Journal of Thrombosis and Haemostasis*.

[15] Goyal J, Adamski J, Lima JL, Marques MB (2013). Relapses of thrombotic thrombocytopenic purpura after treatment with rituximab. *Journal of Clinical Apheresis*.

